# Supplementary material for: AI-driven discovery of novel extracellular matrix biomarkers in pelvic organ prolapse
Source: PLoS Comput Biol. 2025 Oct 7;21(10):e1013483. doi: 10.1371/journal.pcbi.1013483 (PMC12503291; doi:10.1371/journal.pcbi.1013483)
Supplement: Attachment — Submitted filename: Response_to_Reviewers.pdf [file pcbi.1013483.s002.pdf]

Dear Editor and Reviewers,

We sincerely thank you for your insightful and constructive feedback on our manuscript, "AI-Driven Discovery of Novel Extracellular Matrix Biomarkers in Pelvic Organ Prolapse." Your comments have been invaluable in guiding substantial revisions, and we believe the manuscript is now significantly improved in both scientific rigor and clarity. Below, we briefly summarize the key revisions made:

- **Model Upgrade:** We have replaced the previous architecture with the ESM-2 model (650 million parameters), which offers enhanced capability to capture complex sequence patterns in proteins. This leads to more robust and biologically meaningful predictions.
- **Expanded and Balanced Dataset:** The model was retrained on a substantially larger dataset comprising 80,000 proteins (40,000 ECM, 40,000 non-ECM), carefully curated to improve generalizability and reduce data bias.
- **Stricter Evaluation Framework:** To rigorously assess performance, we introduced an independent test set of 16,000 proteins. While overall accuracy slightly decreased (from 99.7% to 99.4%) under this more stringent setting, the model maintained high performance with F1-score = 99.4% and both AUROC and AUPRC = 0.999.
- **Improved Interpretability and Clinical Relevance:** Leveraging the enhanced model, we identified 40 ECM-related proteins from clinical proteomic data, including 24 novel candidates with potential relevance to POP. We also refined the interpretability analysis to provide deeper insights into sequence-level determinants of ECM function.

All major revisions are clearly marked in blue in the revised manuscript for your convenience. We now provide a detailed point-by-point response to each of the reviewer’s comments.

<u>**Reviewer 1**</u>

1. The manuscript mentions rigorous manual curation of the positive dataset. However, it lacks specific details on the criteria used for this curation. Clarifying the selection process and the qualifications of the curators would enhance transparency.

Thank you for raising this important issue. In the revised manuscript (lines 138-151), we now provide a detailed explanation of the positive dataset construction process. We initially

retrieved 40,155 ECM proteins based on GO: 0031012. This list was refined using a two-step curation strategy: Computational filtering for reviewed UniProt entries with high-confidence experimental evidence; Expert manual review by a senior cell biologist with domain expertise in ECM biology, who reviewed UniProt entries and supporting literature to confirm each protein's secreted nature and ECM-related function. This process ensured that only experimentally validated, biologically relevant ECM proteins were included. From the curated list, 40,000 proteins were randomly selected as the final positive dataset.

2. The criteria for selecting the negative dataset (46,427 proteins) are not fully explained. While it mentions the use of SignalP and TMHMM to exclude certain proteins, a more detailed rationale for these exclusions would be beneficial. For instance, why were these specific tools chosen, and how do they relate to ECM protein characteristics?

We appreciate your valuable suggestion. In the revised manuscript (1ines 152-160), clearly explaining the rationale behind using SignalP and TMHMM. We initially collected 46,427 proteins from UniProt that lacked any ECM-related GO annotations. To further minimize the inclusion of any potentially overlooked ECM components, we applied an additional two-step filtering procedure. We chose SignalP because it effectively predicts signal peptides, which typically indicate that a protein is destined for secretion into the extracellular space. Since true ECM proteins are inherently extracellular, excluding proteins with predicted signal peptides helps ensure our negative dataset comprises primarily non-secreted, non-ECM proteins, reducing the risk of misclassifying actual ECM components. TMHMM predicts transmembrane domains. Many cell membrane proteins possess such domains and can interact with the ECM. However, our focus is on predicting proteins that are components of the ECM structure or its direct regulators, not just membrane-bound proteins that interact with it. By removing proteins predicted to have transmembrane domains, we ensured our negative dataset largely consisted of clear-cut intracellular or non-membrane-associated proteins. This combined filtering approach enabled us to create a stringent negative dataset, enhancing the clarity of our training data and mitigating potential biases during model training. Finally, from the curated list, 40,000 proteins were randomly selected as the final negative dataset.

3. The use of CD-HIT with a 30% sequence identity threshold is a common practice, but the implications of this threshold on the diversity of the dataset should be discussed. A

justification for this specific threshold and its impact on model training would provide clarity.

Thanks for prompting us to elaborate here. In the revised manuscript (lines 161-171), we have added a discussion about using CD-HIT with a 30% sequence identity threshold. Picking a 30% sequence identity threshold for CD-HIT is a widely accepted standard in protein bioinformatics, and its main goal is to strike a balance between cutting down on redundant sequences and keeping enough diverse biological information. This threshold effectively groups very similar sequences while still making sure our dataset represents a good variety of protein families, thus avoiding our model getting overly specialized on minor variations within very similar proteins. Ultimately, this helps the model learn generalized features that truly indicate whether a protein is ECM or non-ECM, rather than just memorizing specific, highly related proteins, leading to much more robust model training and a fairer assessment of its classification performance by preventing inflated metrics from nearly identical sequences showing up in both training and test sets.

4. The two-stage transfer learning approach is innovative, but the manuscript should elaborate on the rationale behind the choice of training parameters (e.g., epochs, batch size, learning rate). How were these parameters determined, and were they optimized through preliminary experiments? Providing this information would allow for better reproducibility.

We appreciate you highlighting the innovative nature of our two-stage transfer learning and asking for more detail on our parameter choices. We've thoroughly addressed this in the revised manuscript (lines 191-255). For our EPOP model, built by fine-tuning the powerful ESM-2 (650M parameters, 33 layers, UR50D), the selection of training parameters (epochs, batch size, AdamW optimizer settings) for our 48,000-sequence training dataset was guided by empirical exploration and preliminary experimentation. Our objective was to balance computational efficiency with robust model performance, while actively mitigating common challenges associated with fine-tuning large pre-trained models, such as catastrophic forgetting and overfitting. The two-stage approach was designed to achieve this: the initial adaptation (4 epochs, batch size 32, frozen ESM-2 layers) quickly adapted the classification head, followed by full fine-tuning (2 epochs, effective batch size 16 via gradient accumulation, adjusted AdamW) for more subtle, specific feature capture. While we did not conduct a formal, exhaustive hyperparameter sweep, these settings were meticulously

determined through extensive preliminary experiments to achieve satisfactory convergence and ensure the reproducibility of our fine-tuning process. Model performance was continuously monitored on a held-out 16,000-sample validation set throughout both stages, with the best-performing checkpoint saved for final evaluation. Similarly, specific training parameters for our baseline Transformer and LSTM models were empirically determined, taking into account practical limitations of the GPU environment, especially the comparatively slower training speed of the LSTM model. These settings were chosen to facilitate effective convergence and robust performance for these deep learning architectures on the protein sequence classification task, and these choices were also informed by preliminary experimentation to ensure satisfactory results and reproducibility.

5. The validation strategy using 20,000 independent sequences is mentioned, but the manuscript does not specify how these sequences were selected or their relevance to the training data. A clearer explanation of the validation set's composition and its relationship to the training data would enhance the robustness of the findings.

We appreciate your request for greater detail on our validation strategy. We've refined the revised manuscript to clearly outline our data partitioning and the role of our independent test set. We started with a meticulously curated and balanced dataset of 80,000 proteins (40,000 ECM and 40,000 non-ECM) for model training and evaluation (lines 135-137). The entire data processing pipeline, including initial raw datasets and subsequent splitting into training, validation, and test sets, is visually outlined in Figure 2. To ensure an unbiased and accurate assessment of our model's generalization, we strategically divided this dataset. After redundancy reduction, the entire dataset was randomly partitioned into training (48,000), validation (16,000), and independent test (16,000) sets (lines 172-178). This random partitioning guarantees each subset represents the overall protein diversity and that the test set remains entirely unseen during training and validation, enabling an unbiased evaluation of generalization performance. During our two-stage transfer learning, we continuously monitored model performance on the 16,000-sample validation set to gauge convergence and generalization, saving the best-performing model checkpoint (lines 224-227). Finally, we evaluated the model's performance on the completely independent 16,000-sample test set (8,000 positive and 8,000 negative), which had no overlap with the training data (lines 334-336). This rigorous and comprehensive data partitioning significantly strengthens the reliability and robustness of our findings.

6. The literature-based validation process is mentioned, but the criteria for selecting the curated list of ECM-related proteins should be detailed. How were these proteins identified, and what sources were used?

Thanks for asking for more specifics about our literature-based validation. We've now detailed this process in the revised manuscript (lines 296-305). To assess the biological relevance and potential novelty of our predicted ECM proteins, we conducted a comprehensive literature review. This involved manual searches using Google Scholar and PubMed, combining general terms like “pelvic organ prolapse”, “extracellular matrix”, “collagen”, “elastin”, “fibronectin,” and “tenascin” with specific protein names or UniProt identifiers from our proteomic predictions. For each protein, we carefully examined retrieved publications to find clear evidence of a functional role or direct association with POP. This systematic review allowed us to classify our predicted ECM proteins into those previously reported in POP literature and novel candidates for the condition. This rigorous approach ensures our claims of novelty for the 24 newly identified proteins are well-supported by current scientific literature, confirming the biological relevance of our findings.

7. The manuscript should discuss potential limitations of the interpretability analyses. For instance, how might the findings be affected by the inherent biases of the model or the datasets used?

We appreciate you highlighting the critical importance of discussing the limitations of interpretability analyses. We’ve added a dedicated discussion on this point in the revised manuscript (lines 651-662). While our interpretability analyses offer really valuable insights, we recognize that they can be affected by biases built into both the model and the datasets. For instance, the ESM-2 model, being pre-trained on a broad protein corpus, may inherently reflect biases present in its vast training data distribution. These biases could influence which specific sequence features are highlighted as important for ECM-relatedness. Similarly, our specific datasets also carry potential biases, such as limitations in UniProt annotations (e.g., incomplete or outdated information) for some proteins, or the characteristics of the POP patient proteomic samples (e.g., sample size, heterogeneity, technical detection limits). These factors might affect the generalizability and robustness of the interpretability findings. So, while our analyses give us great initial insights into what makes ECM proteins tick, we need to keep these inherent data and model biases in mind when we talk about the exact sequence determinants, and further validation in different contexts is definitely needed.

8. The manuscript suggests that the EPOP framework could be extended to other ECM-related disorders. However, it should address how the model's findings might generalize to different contexts or populations. Are there specific considerations or adaptations needed for applying this model to other diseases?

We appreciate you prompting us to think about the broader applications of our EPOP framework. We've expanded on this in the revised manuscript (lines 702-713), covering how our model might generalize to other ECM-related disorders and what adaptations would be needed. Our EPOP framework, built on the solid ESM-2 model, definitely shows strong potential for tackling other diverse ECM-related disorders, including fibrotic diseases, cardiovascular conditions involving ECM remodeling, and certain cancers. However, applying the model to different disease contexts or populations isn't a simple plug-and-play; it requires careful thought and adaptation: ECM structure and molecular mechanisms can change quite a bit from one disease to another, so a model trained primarily on POP data might not directly apply. Factors like a person's genetic background, age, and ethnicity can all influence ECM makeup and how a disease shows up, so if we apply the model to diverse groups beyond our initial POP patient cohort, we'll need thorough validation. For new applications, we'd need to fine-tune EPOP's classification head (and possibly certain layers of the pre-trained ESM-2 backbone) using high-quality proteomic datasets specific to that new disease. This targeted fine-tuning is key to help the model pick up on the ECM protein characteristics relevant to the new disease. On top of that, bringing in other relevant biological information (like clinical data or genetic markers) and doing rigorous experimental validation would be essential to confirm the accuracy and clinical utility of predictions in these novel applications. By carefully taking these adaptation and validation steps, the EPOP framework could become a really versatile tool for systematically discovering disease mechanisms and therapeutic targets across a wide range of ECM-related conditions.

Reviewer 2

1. 99.7% accuracy is repeatedly emphasized. This is extremely high and may not generalize to real-world datasets. There's no test on independent, external datasets (e.g., from another tissue, experiment, or species). Include external validation to test generalizability or tone down the performance claims.

We totally agree with this crucial point. Our initial manuscript indeed emphasized a very high accuracy, and you're right to question its generalizability without more rigorous, independent

validation. We’ve made significant changes to address this concern directly in the revised manuscript. Our new accuracy is 99.40%, which is still exceptionally high but reflects a more robust and balanced evaluation under stricter conditions. We no longer solely emphasize a single accuracy figure. Our evaluation is now explicitly based on a 16,000-sample independent test set (8,000 positive and 8,000 negative samples), completely unseen during training, providing a much more reliable assessment of generalizability (lines 333-374). We've also introduced a new Figure 4 that offers a far more detailed and nuanced view of our model's performance, including Precision-Recall Curve (AUPRC = 0.999), ROC Curve (AUROC = 0.999), score distributions, confusion matrix, and performance across various thresholds, highlighting the optimal F1-score threshold of 0.168. To apply EPOP to real-world proteomic data, we implemented a robust pipeline for peptide-level prediction, multi-step protein identification, and functional enrichment analysis (lines 256-308). We applied our EPOP model to a proteomic dataset (PRIDE: PXD011467) derived from POP patient vaginal wall tissues. This serves as a vital form of external validation, demonstrating the model's practical utility and generalizability to a clinical context. Our success in identifying numerous known and novel ECM proteins within this dataset directly supports the model's applicability beyond its training data.

2. No wet-lab or histological validation for novel biomarkers (e.g., EMILIN-1). Acknowledge this limitation more clearly in the discussion. Suggest an experimental pipeline for validation.

We fully agree with your insightful comment regarding the need for experimental validation of novel biomarkers such as EMILIN-1. Although we had previously noted this limitation (Lines 635–639), we appreciate the suggestion to state it more explicitly. This limitation is indeed significant and represents a critical next step toward translating our computational findings into clinically actionable insights. To guide future work, we’ve laid out a detailed experimental pipeline in the revised manuscript (lines 670-681) that outlines how these novel biomarkers can be validated: (i) immunohistochemical staining of pelvic support tissues from POP patients and healthy controls to assess spatial expression patterns; (ii) quantification of protein levels using Western blot on tissue lysates or biological fluids; (iii) in vitro functional assays using mechanically stressed fibroblasts, with knockdown or overexpression of target proteins, to evaluate their role in ECM remodeling; and (iv) in vivo validation using animal models of pelvic floor dysfunction to assess their impact on tissue integrity and disease progression. We believe this comprehensive, multi-level experimental approach will be

essential to establish the mechanistic roles of these proteins in POP pathogenesis and determine their suitability as reliable biomarkers or therapeutic targets.

3. Negative class = "proteins lacking ECM-related GO terms" and filtered by SignalP/TMHMM. This may introduce annotation bias and exclude many unknown ECM-related proteins from the negative set. Add a discussion about potential bias and the implications on classification fairness.

Thank you for highlighting this important issue regarding potential biases in the construction of the negative dataset. We fully acknowledge this challenge and have added a dedicated discussion in the revised manuscript (Lines 640–650) to address it more explicitly. We explain that while our strategy of defining non-ECM proteins based on the absence of ECM-related GO annotations, followed by SignalP and TMHMM filtering, certainly helps to clarify labels, it does come with a risk of annotation bias. Here's why: proteins that are genuinely ECM-related but lack comprehensive or up-to-date annotations (for example, newly discovered ECM regulators or unusual secreted factors) might have been mistakenly included in our "negative" set. If such mislabeled proteins were present in the negative training set, the model might be "penalized" during training for correctly identifying them as ECM components, even though our intent was to train it on clear non-ECM examples. This could potentially limit the model's ability to accurately generalize to unannotated or unconventional ECM components in real-world scenarios. We highlight this as a fundamental challenge in supervised learning when relying on imperfect biological annotations.

4. A threshold of 0.6 for peptide classification is used without statistical justification.

We appreciate your request for a clear statistical justification of our classification threshold. We've clarified this crucial detail in the revised manuscript. We no longer use an arbitrary 0.6 threshold. Instead, the threshold for classifying peptides as high-confidence ECM-related was statistically optimized to 0.168. This specific value was determined on our independent validation dataset by maximizing the F1-score, which is a standard metric that balances both precision and recall, essential for robust classification in biological contexts (lines 266-269). This optimization is further reinforced by our analysis of model performance across varying classification thresholds and cumulative score distributions (lines 375-382), clearly illustrating how the 0.168 threshold maximizes the F1-score and emphasizes the clear separation in prediction confidence

5. Discussion of novel proteins (e.g., EMILIN-1, G protein subunit beta-1) is too brief and speculative.

We completely agree that the discussion of our novel protein findings needed more depth. We have significantly expanded this part in the revised manuscript (lines 445-491). In the revised manuscript, we've provided a much more comprehensive analysis of the 24 novel ECM proteins identified by EPOP. Instead of just briefly mentioning a few, we now discuss them as a group in Table 3, providing their UniProt IDs, gene names, and detailed functional annotations. For key examples like POSTN (Q15063), NID2 (Q14112), SOD3 (P08294), and proteins involved in immune/inflammatory pathways (e.g., CMA1, C3, CFB, CFD), we've elaborated on their known biological roles and plausible connections to ECM integrity, cell adhesion, tissue remodeling, immune modulation, or inflammation. We also briefly discuss proteins involved in oxidative stress response (SOD3) and vesicle transport (RAB1A, RAB1B), linking their general functions to potential indirect effects on ECM. This expanded discussion moves beyond mere speculation, grounding the potential relevance of these novel proteins in their established biological functions as documented in UniProt and other literature. We explicitly state that these findings provide a valuable foundation for future mechanistic investigations, positioning them as promising novel candidates for POP research beyond currently known associations. We hope this provides a more robust and informative context for these exciting new discoveries.

6. Was k-fold cross validation used during training? Please specify.

Thanks for asking for this important clarification. No, we didn't use k-fold cross-validation during the training of EPOP or our baseline models. Instead, we adopted a single, robust data split strategy for our final model training and evaluation. Here's how we did it: We started with a balanced dataset of 80,000 proteins (lines 135-137). trained our model on a prepared training dataset of 48,000 sequences. Throughout training, model performance was continuously monitored on a held-out validation set of 16,000 samples (lines 197-223). Crucially, the final, unbiased performance evaluation was conducted using a completely independent test set of 16,000 samples (8,000 positive and 8,000 negative) that didn't overlap with the training data (lines 334-336). This comprehensive approach ensured our model's performance was assessed on data it had never seen, providing a clear indication of its generalization capabilities. While k-fold cross-validation is valuable, we believe our chosen

method with a large, independent test set provides sufficient rigor for the reported performance claims.

Reviewer 3

1. There are minor typographical errors in the manuscript at several points, including Figure 4 caption, lines 142, 263, 285, and 305.

Thank you for your careful review and valuable feedback. We appreciate you pointing out the minor typographical errors in the manuscript. We have thoroughly reviewed the manuscript and corrected all the identified errors. We have also performed an additional proofread of the entire manuscript to ensure no other similar errors remain.

2.. Threshold Selection Rationale (Line 142): The manuscript mentions a confidence threshold of 0.6 for ECM classification. While this value appears reasonable, the rationale behind its selection is not provided. I recommend specifying whether this threshold was chosen based on a particular statistical criterion, such as ROC curve optimization, Youden's index, or empirical tuning on a validation set.

Thanks for highlighting the need for statistical justification regarding our classification threshold. You're absolutely right to ask for this crucial detail. We've clarified this in the revised manuscript. We no longer use an arbitrary 0.6 threshold. Instead, the threshold for classifying high-confidence ECM-related peptides was statistically optimized to 0.168 (lines 266-269). This specific value was determined on our independent validation dataset by maximizing the F1-score, a standard metric that balances both precision and recall, which is essential for robust biological classification.

3. Performance Evaluation of ECM Function Prediction Models (Lines 179-206): The comparative performance analysis of the ECM function prediction models is informative. However, in cases like the LSTM model, where recall is high but precision is low, the inclusion of the F1-score would provide a more balanced view of model performance. Please consider adding this metric to Table 1 or the main text for completeness.

We couldn't agree more with this suggestion. Adding the F1-score is essential for a more balanced and complete picture of model performance, especially when dealing with varying precision and recall values. In the revised manuscript, we've updated Table 1 to include the F1-score for all models. As detailed in the text (lines 349-357), EPOP achieved an

outstanding F1-score of 99.40%, indicating an excellent trade-off between precision (99.50%) and recall (99.30%). In contrast, the Transformer's F1-score was 87.74%, reflecting a disparity between its high precision and moderate recall, while the LSTM, despite achieving a decent recall, showed lower precision leading to an F1-score of only 79.04%. This comprehensive analysis now clearly demonstrates EPOP's superior ability to maintain high performance across multiple critical metrics compared to baseline methods.

4. Prediction Results of POP-Associated ECM Proteins (Lines 207-249):

4.1 The phrase "no significant change" should be clearly defined. Was this determined by a fold-change cutoff, a p-value threshold, or another statistical test? Including this information will improve the interpretability of the results.

We appreciate you highlighting the need for clear definitions regarding expression changes. You are absolutely right that such terms require precise context. In the revised manuscript, we've refined how we present protein expression patterns in Table 2. We no longer provide a blanket definition for "no significant change". Instead, when referring to expression levels like "downregulated" "upregulated" or "no significant change" for specific proteins, these patterns are directly attributed to the original cited literature, and other references in Table 2. These original studies would have used their specific statistical criteria (such as fold-change cutoffs or p-value thresholds) to determine significance. Our primary focus with EPOP is the identification and ECM-relatedness of proteins based on their sequence, while the detailed differential expression analysis rests on the findings reported in the respective original publications.

4.2 When noting that certain proteins "were not previously reported", it would be helpful to clarify whether this determination was made through a systematic literature review, database query (e.g., DisGeNET, PubMed), or both.

We appreciate you asking for clarification on how we determined that certain proteins were not previously reported in the context of POP. You are absolutely right that this process needs to be transparent. This determination was made through a systematic literature review, as described in detail in the revised manuscript (lines 296-305). Our process involved:

- Extensive database queries: We searched PubMed and other relevant biomedical databases using comprehensive keyword combinations related to "pelvic organ

prolapse" and extracellular matrix components, as well as specific protein names and UniProt IDs.

- Manual review: Our team manually reviewed the search results to ascertain if a direct, explicit association or functional study in the context of POP had been published for each protein.

This systematic approach ensured that our claims of novelty for the 24 newly identified proteins (introduced in lines 445-491 and clearly listed in Table 3) are rigorously supported by the current scientific literature.

We sincerely hope the revised manuscript meets your expectations. Thank you once again for your time and invaluable feedback.
