# Supplementary material for: AI-driven discovery of novel extracellular matrix biomarkers in pelvic organ prolapse
Source: PLoS Comput Biol. 2025 Oct 7;21(10):e1013483. doi: 10.1371/journal.pcbi.1013483 (PMC12503291; doi:10.1371/journal.pcbi.1013483)
Supplement: Attachment — Submitted filename: Comments to the authors.docx [file pcbi.1013483.s001.docx]

**Comments to the authors:**

This study introduces EPOP (Extracellular Matrix Protein Predictor), a deep learning framework specifically designed for ECM protein classification and disease mechanism discovery. The authors apply a sophisticated two-stage transfer learning approach using the ESM-2 protein language model, fine-tuned on large curated datasets comprising over 86,000 proteins. The model integrates attention mechanisms and interpretability modules and is rigorously validated using 20,000 independent sequences and clinical proteomics data, with a focus on pelvic organ prolapse (POP) as a clinically relevant application.

EPOP achieves impressive classification accuracy of 99.7%, significantly outperforming existing Transformer and LSTM architectures. Beyond performance, the model identifies 10 novel disease-associated ECM proteins, including EMILIN-1, and reveals new patterns of ECM remodeling in disease. Importantly, interpretability analyses provide biological insights into sequence motifs and structural features critical to ECM function. Ablation studies further confirm the robustness of the architecture.

The manuscript is well-written and presents a thoughtful approach to identifying POP-associated ECM proteins. The study is methodologically sound, and the results are compelling, with only a few minor clarifications needed for improved transparency.

**Minor Comments and Suggestions:**

1. There are minor typographical errors in the manuscript at several points, including Figure 4 caption, lines 142, 263, 285, and 305.
2. **Threshold Selection Rationale (Line 142):** The manuscript mentions a confidence threshold of 0.6 for ECM classification. While this value appears reasonable, the rationale behind its selection is not provided. I recommend specifying whether this threshold was chosen based on a particular statistical criterion, such as ROC curve optimization, Youden's index, or empirical tuning on a validation set.
3. **Performance Evaluation of ECM Function Prediction Models (Lines 179-206):** The comparative performance analysis of the ECM function prediction models is informative. However, in cases like the LSTM model, where recall is high but precision is low, the inclusion of the F1-score would provide a more balanced view of model performance. Please consider adding this metric to Table 1 or the main text for completeness.
4. **Prediction Results of POP-Associated ECM Proteins (Lines 207-249):**
   - The phrase "no significant change" should be clearly defined. Was this determined by a fold-change cutoff, a p-value threshold, or another statistical test? Including this information will improve the interpretability of the results.
   - When noting that certain proteins "were not previously reported", it would be helpful to clarify whether this determination was made through a systematic literature review, database query (e.g., DisGeNET, PubMed), or both.
